# Automating Speech Audiometry in Quiet and in Noise Using a Deep Neural Network

**DOI:** 10.3390/biology14020191

**Published:** 2025-02-12

**Authors:** Hadrien Jean, Nicolas Wallaert, Antoine Dreumont, Gwenaelle Creff, Benoit Godey, Nihaad Paraouty

**Affiliations:** 1 R&D Department, My Medical Assistant SAS, 51100 Reims, France; 2 Department of Otorhinolaryngology-Head and Neck Surgery, Rennes University Hospital, 35000 Rennes, France; 3 Audition Dreumont Clinic, 56100 Lorient, France

**Keywords:** speech audiometry, automated speech recognition, machine learning, speech-in-noise, deep neural network

## Abstract

In addition to pure-tone audiometry tests and electrophysiological tests, a comprehensive hearing evaluation includes assessing a subject’s ability to understand speech in quiet and in noise. In fact, speech audiometry tests are commonly used in clinical practice; however, they are time-consuming as they require manual scoring by a hearing professional. To address this issue, we developed an automated speech recognition (ASR) system for scoring subject responses at the phonetic level. The ASR was built using a deep neural network and trained with pre-recorded French speech materials: Lafon’s cochlear lists and Dodelé logatoms. Next, we tested the performance and reliability of the ASR in clinical settings with both normal-hearing and hearing-impaired listeners. Our findings indicate that the ASR’s performance is statistically similar to manual scoring by expert hearing professionals, both in quiet and in noisy conditions. Moreover, the test–retest reliability of the automated scoring closely matches that of manual scoring. Together, our results validate the use of this deep neural network in both clinical and research contexts for conducting speech audiometry tests in quiet and in noise.

## Simple Summary

Understanding speech in noisy environments is a challenge for everyone. Therefore, it is crucial to assess speech-in-noise intelligibility in a range of listening environments. Hearing evaluations often include speech intelligibility tests, known as speech audiometry tests, conducted in both quiet and noisy settings. However, these tests are time-consuming as hearing specialists need to listen to and manually score patient verbal responses. To solve this problem, we developed an automated system that uses specially designed speech recognition algorithms to evaluate patient verbal responses automatically. This system is based on artificial intelligence, more precisely, a deep neural network that has been trained with pre-recorded speech audiometry materials. We tested this automated system in real-world clinical settings and found that it scored patient responses just as accurately as human experts, both in quiet and in noisy conditions. Furthermore, the results were consistent when the tests were repeated, showing the system’s reliability. In summary, this automated system offers an alternative to manual scoring of speech audiometry tests, saving time for hearing professionals while maintaining the level of accuracy and test–retest reliability.

## 1. Introduction

Today, clinical hearing assessments primarily rely on pure-tone audiometry, which is traditionally conducted manually by a healthcare practitioner [1]. Pure-tone audiometry is a non-invasive test and involves measuring the detection thresholds of pure tones through air and bone conduction, using a modified version of the Hughson–Westlake procedure [2,3]. However, for most individuals, the initial symptom of hearing loss is difficulty perceiving speech sounds, particularly, in noisy environments [4,5].

A recent study [6] examined over 100,000 records from the Massachusetts Eye and Ear audiology database and the authors showed that approximately 10% of patients reporting hearing difficulties were sent home with a “clean bill of hearing health” based on “normal” pure-tone audiometry results. These findings highlight the critical need for supplementary tests in hearing assessments—tests that better reflect complex, real-world environments rather than solely detecting the softest pure tones at different frequencies (i.e., pure-tone audiometry). One such test is speech audiometry [7,8,9,10,11]. In fact, speech audiometry is widely used in most countries as part of comprehensive hearing evaluations (e.g., [12,13]). Additionally, it plays a crucial role in auditory evaluations for hearing aid assessments and fittings [14].

Speech audiometry assesses a patient’s ability to understand words by varying either the intensity of the sound (for speech audiometry conducted in quiet) or the intensity of accompanying background noise (for speech audiometry conducted in noise). This evaluation considers both peripheral auditory factors and higher-level cognitive processes, including language comprehension, attention, and decision-making abilities [7,15,16]. A common metric used in speech audiometry is the speech reception threshold (SRT), defined as the signal presentation level (in dB for speech-in-quiet) or signal-to-noise ratio (SNR, in dB, for speech-in-noise) at which 50% speech intelligibility is achieved. Further key indicators of speech recognition include the maximum speech intelligibility score (in %) and the slope of the psychometric function at 50% intelligibility.

Although most hearing professionals today rely on pre-recorded auditory tests for speech audiometry [17], its overall application remains limited due to constraints in time and equipment (as reviewed in [18]). A fundamental challenge in the application of speech audiometry lies in linguistic, phonetic, and melodic barriers, as well as variations in accents and intonation. The auditory requirements for understanding speech differ significantly across languages, making it particularly challenging for healthcare practitioners whose native language differs from that of the test material. Over the past few decades, advancements in speech audiometry techniques have addressed some of these challenges. This progress includes the development of diverse speech materials, including “open” speech lists validated in multiple languages (e.g., [19,20,21]). Furthermore, the introduction of digit tests (e.g., [22,23]) and speech materials featuring logatoms and meaningless words (e.g., [24,25]) have in part mitigated linguistic requirements.

A crucial issue with current testing methods is the necessity of a human expert to conduct the test and record the patient’s responses or errors throughout the entire speech audiometry assessment. Phoneme or word scoring is performed manually by the expert which is a time-intensive process (approximately, 10–20 min for each speech audiometry test [26]). To address this, automated methods have been proposed (e.g., AMTAS [27]). However, automated speech audiometry remains limited to forced-choice tests (i.e., closed-set formats [27,28]), where the patient selects an answer from a predefined list of options. This approach restricts the ability to analyze phonetic confusions and, in some cases, relies on speech lists or materials that are not standardized (e.g., [29]). Briefly, standardized lists are word lists from the subject’s everyday lexical field with the same phoneme occurrences as in the language, and phonetically balanced (in terms of vowels and consonants) within or across the word lists.

In order to save time for hearing professionals, automated speech recognition (ASR) systems have recently been developed, which allows the automation of speech audiometry tests (e.g., [30,31,32]). Ooster and colleagues [33] made a significant contribution to the field by showcasing the potential of machine learning (ML) in speech audiometry. Their work provides foundational evidence for the feasibility of ML-driven speech audiometry tests, showing that an ASR system could achieve 50% intelligibility scores within the range of human test–retest accuracy. While a variety of ASR-based speech-hearing tests are available for non-clinical use, for example, smartphone applications [34,35,36], their performances in home settings do not reach clinical standards [37,38]. To address this gap, Ooster and colleagues [39] developed a self-administered speech audiometry test for use at home with “smart” speakers (i.e., voice-controlled audio devices with a virtual assistant). For clinical applications, an efficient ASR system for the digit-in-noise test [40] was developed and tested with both normal-hearing and unaided hearing-impaired listeners [41]. Similarly, Araiza-Illan and colleagues [42] employed an open-source ASR algorithm [43] to automatically score the digit-in-noise test. Despite these advancements (see review [44]), the use of ASR for capturing and automatically scoring verbal patient responses during speech audiometry remains quite limited in clinical practice today (only seven studies are reported in [44]).

In the current study, we developed and implemented an ASR algorithm within the audiometry software iAudiogram^®^ (v1; My Medical Assistant SAS, Reims, France) to enable fully automated speech audiometry tests in quiet, with Lafon’s cochlear lists [45], and in noise with Dodelé logatom lists [46]. Both speech materials consist of open speech lists that are commonly used in daily clinical practice in France [13,18,47]. The software iAudiogram^®^ has recently been validated in two former studies for fully automated air-conduction [48] and bone-conduction [49] pure-tone audiometry tests. In this study, for speech audiometry, the ASR algorithm developed enables automated logging and phonetic-level scoring of subject responses. Leveraging recent advances in machine learning (ML), this approach aims to save time for hearing professionals (through automation), while maintaining accuracy and test–retest reliability by eliminating human errors and variability.

First, we evaluated the performance of the ASR system on a large sample of normal-hearing (NH) and unaided hearing-impaired (HI) subjects by comparing the automated scores with manual scores recorded by expert hearing professionals for both tests. Next, we analyzed the test–retest reliability of both the automated and manual scoring methods. Together, our results suggest that automated speech audiometry tests can be routinely integrated into daily clinical practice without requiring as much time and involvement of a hearing practitioner, as the speech audiometry tests are fully automated. Ultimately, the adoption of ASR systems could promote a more systematic use of speech audiometry tests, especially, speech-in-noise tests, during hearing evaluations by healthcare professionals (e.g., French national health insurance statistics for year 2023: 398,456 pure-tone tests vs. 98,364 speech-in-noise tests). Such systematic use of a more comprehensive assessment of hearing health may contribute to the timely detection of hearing loss, thereby limiting the development or worsening of accompanying comorbid conditions, such as social isolation and depression.

## 2. Materials and Methods

### 2.1. Automated Speech Recognition (ASR) System

Our goal was to develop a deep neural network (DNN), pre-trained on sound samples, to transcribe phonemes/words uttered by the patient, in order to determine whether the phonemes/words were correctly heard and repeated by the patient. For speech audiometry in quiet, we used Lafon’s Cochlear list in French language. For speech audiometry in noise, we used Dodele Logatoms in French language. We opted for phoneme-level scoring instead of sentence-level scoring to reduce the overall testing time. In other words, presenting words/logatoms and using phoneme-level scoring provided more information in a shorter time compared to presenting sentences and using sentence-level scoring. Existing ASRs (e.g., from IBM and Google) are not designed for phoneme identification and rely heavily on linguistic and contextual information, making them unsuitable for speech audiometry. Since linguistic and contextual data are absent from most speech audiometry material, particularly for phoneme recognition, we needed to develop an ASR specifically tailored for this purpose.

The ASR model was built using the wav2vec 2.0 model [50], a pre-trained model based on self-supervised learning, which allows data representations to be learned from unlabeled data. This approach is similar to models used in natural language processing, such as Bert [51]. Next, the ASR model was fine-tuned through supervised learning using anonymized speech audiometry data [52]. More precisely, speech audiometry data (i.e., patient verbal responses) from three audiology clinics in France (located in Lorient, Reims, and Rennes) were recorded and manually scored at both the phonemic and word levels by more than 10 hearing healthcare professionals, including the co-authors AD, NW, and GC.

The training database consisted of 23,995 word samples of Lafon’s cochlear lists that were correctly repeated by patients and 3028 word samples of Dodele Logatoms. An example of fine-tuning for a speech recognition task is provided in [50], where the model takes raw audio word samples as input and outputs alphabetic characters. In our case, we trained the model to take audio word samples as input and output a sequence of phonemes. Specifically, a predefined list of phonemes was provided (these are the phonemes the model can output), along with an ‘unknown’ token for cases where the model cannot identify the phoneme, and a ‘blank’ token [53] for cases where the model detects no phoneme being pronounced.

To assess whether the outputted phoneme sequence is correct, we next compare it with the “ground truth” phoneme list, which was manually scored by the hearing healthcare professionals. Since the alignment between the input audio samples and the output phonemes is unknown, we used a connectionist temporal classification (CTC) cost function [51,53]. This comparison is made at the phoneme level. This approach mirrors one of the experiments conducted in [50], in which the authors fine-tune a model for phoneme recognition using the TIMIT database and achieve state-of-the-art scores. The cost function is calculated by comparing an audio segment containing several phonemes with a target sequence that also consists of several phonemes.

To pre-validate the ASR performance with Lafon’s cochlear lists, we compared it against 8094 samples (not used during training). All the samples used in this pre-validation were correctly repeated by the patients. An ideal model would achieve a word accuracy and a phoneme accuracy of 1.00. The tested ASR achieved a word accuracy of 0.948 and a phoneme accuracy of 0.979. Next, we used a different set of 8094 samples (also not used during training), but this time the samples were incorrectly repeated by the patients. An ideal model would have a word and a phoneme error rate of 0. The ASR tested achieved a word error rate of 0.012 and a phoneme error rate of 0.174.

Similarly, to pre-validate the ASR performance with Dodelé logatoms, we compared it against 1020 samples (not used during training) that were all correctly repeated by the patients. The ASR achieved a word accuracy of 0.925 and a phoneme accuracy of 0.964. With a different set of 1020 samples (also not used during training), that were incorrectly repeated by the patients, the ASR tested achieved a word error rate of 0.012 and a phoneme error rate of 0.161. Overall, for both speech materials, those pre-validation scores were deemed sufficient to proceed to the actual validation phase in clinical settings.

### 2.2. Subjects

The study was approved by the French Regional Ethics Committee (Comité de Protection des Personnes Est III; SI number: 22.03364.000107) and all methods were performed in accordance with the relevant guidelines and regulations. All subjects were recruited from three audiology centers in France, located in Lorient, Reims, and Rennes over a 6-month period. Three expert hearing professionals (1 ENT (GC) and 2 audiologists (AD and NW); with former otology training and clinical expertise of more than 5 years) conducted all tests (i.e., pure-tone and speech audiometry tests), including manual scoring of subjects’ verbal responses.

Subjects were fully informed of the goal of the study and provided written informed consent before their participation. No financial compensation was provided as all tests were performed either during the initial or follow-up hearing assessment. To ensure a balanced population of subjects, previous hearing status (when available) was used to recruit subjects with varying levels of hearing loss severity. All subjects included in the study had French as their mother tongue, good diction, and no strong accent (confirmed by the experimenters prior to inclusion of subject in study using a short oral discussion).

All subjects were instructed to clearly repeat the word they believed they had heard upon seeing a green light on a monitor placed in front of them. Subjects were informed that they had 3 s after the green light to repeat the word (words repeated before the green light or after the 3 s were systematically rejected). Subjects were also informed that the intensity of stimulation and/or masking noise could vary and some words, including meaningless words, would be difficult to hear. In summary, they were instructed to simply repeat the words they heard and refrain from saying additional phrases, such as “I don’t know” or “I am not sure”.

#### 2.2.1. Subjects for Speech Audiometry in Quiet

A group of 109 subjects was tested (52 men, 57 women; 18–94 years; 69.2 ± 18.9 years). For speech audiometry in quiet, each of the two ears of a given subject was tested individually using Radioear DD450 headphones (Middelfart, Denmark). The pure-tone average (PTA, for Methods, see Section 2.4) computed for each ear revealed a distribution of hearing status as follows:

- Normal hearing with PTA less than 20 dB (n = 37 ears);
- Mild hearing loss with PTA between 21 and 40 dB (n = 49 ears);
- Moderate hearing loss with PTA between 41 and 70 dB (n = 126 ears);
- Severe hearing loss with PTA between 71 and 90 dB (n = 6 ears).

#### 2.2.2. Subjects for Speech Audiometry in Noise

A group of 185 subjects was tested (including a subset of subjects tested in quiet; 92 men, 93 women; 18–86 years; 47.3 ± 24.5 years). For speech audiometry in noise, both ears of a given subject were tested together in free-field using Focal Sib loudspeakers. The mean pure-tone average (PTA, for Methods, see Section 2.4) computed for both ears of each given subject revealed a distribution of hearing status as follows:

- Normal hearing with PTA less than 20 dB (n = 85 subjects);
- Mild hearing loss with PTA between 21 and 40 dB (n = 50 subjects);
- Moderate hearing loss with PTA between 41 and 70 dB (n = 50 subjects).

### 2.3. Material and Calibration

All testing took place in an audiometric booth. The audiometer used for pure-tone audiometry was a Natus Aurical audiometer (Pleasanton, CA, USA). TDH39 headphones (Telephonics, Huntington, NY, USA) mounted on Peltor earmuffs were used for pure-tone audiometry tests. All hearing-impaired (HI) subjects were tested without their hearing aids and without any compensation for sound or noise presentation levels.

The automated speech audiometry was performed with the software iAudiogram^®^ (v1; My Medical Assistant SAS, Reims, France). All stimuli were generated at a sampling frequency of 48 kHz and a resolution of 24 bits. The digital-to-analog conversion was performed by an audio interface (Willich, Germany) without acoustic attenuation.

Speech audiometry in quiet was performed with Radioear DD450 headphones (Middelfart, Denmark) positioned securely on the ears of subjects. Speech audiometry in noise was performed in free-field, with four Focal Sib loudspeakers (amplifiers: TPA3116) positioned 1.25 m around the subject. The stimuli came from the speakers positioned at azimuth +45° and −45°. The noise was presented from the remaining 2 speakers positioned at azimuth +135° and −135°.

Calibration was performed by a specialist technician in accordance with EN IEC 60318-1:2009, using a Brüel & Kjær 4153 coupler (Copenhagen, Denmark), a Brüel & Kjær 0843 adaptor (Copenhagen, Denmark), and a Brüel & Kjær 0304 cone (Copenhagen, Denmark). The sound pressure level was measured with a Brüel & Kjær 2250 sound level meter (Copenhagen, Denmark).

### 2.4. Manual Air-Conduction Pure-Tone Audiometry to Obtain the Hearing Status of Subjects

The hearing status of all subjects was assessed using the pure-tone audiometry performed by the experimenters (i.e., the three expert hearing professionals). Manual air-conduction (AC) pure-tone audiometry test systematically begins by testing the better ear declared by the patient, and if no better ear was declared, the right ear was tested first. The manual audiometry procedure tests audiometric frequencies of 1, 1.5, 2, 3, 4, 6, 0.75, 0.5, and 0.25 kHz in the given order as recommended in different audiometry guidelines [54,55]. The intensity level varied in 5 (up) and 10 dB steps (down), also referred to as an asymmetric up–down procedure [56]. Contralateral masking was used, when necessary, in line with [20]. A pure-tone average (PTA) was computed for each subject by averaging AC threshold measures at the following frequencies: 500, 1000, 2000, and 4000 Hz [57]. Hearing status was obtained from the PTA measures [57]: normal hearing (NH): PTA less or equal to 20 dB; mild hearing loss: PTA between 21 and 40 dB; moderate hearing loss: PTA between 41 and 70 dB; and severe hearing loss: PTA between 71 and 90 dB.

### 2.5. Speech Audiometry in Quiet with Lafon’s Cochlear Lists

The experimenters always started by testing the ‘better ear’ of the subject from their pure-tone audiometry results. The stimuli presentation level (PL) varied between 0 to 90 dB HL, by 10 dB steps. An initial Lafon’s cochlear list was randomly chosen from the 20 Lafon’s cochlear lists (17 words of 3 phonemes each) and the first PL was fixed at 20 dB above the PTA (if the PTA was <60 dB). If the PTA was between 60 and 80 dB, the first PL was fixed at 10 dB above the PTA. Finally, if the PTA was above 80 dB, the first PL was fixed at 5 dB above the PTA. All 17 words from a given list were tested at each stimulus PL. If the score obtained after the first 7 words was less than 15%, the list was stopped, the intelligibility score obtained was saved, and the next audiometry point (i.e., 10 dB lower) was then automatically set to 0% correctly perceived phonemes, without being tested. If 100% intelligibility has not been achieved, the PL was set 10 dB above the first point tested, until 100% intelligibility is achieved or the value of the derivative of the psychometric function reversed (i.e., decreased), indicating the appearance of a speech maximum. Above a PL of 80 dB HL, the step is reduced from 10 to 5 dB to guarantee that hearing-impaired (HI) subjects are not presented with excessively high sound levels. This limits auditory overstimulation and uncomfortable levels that could occur due to loudness recruitment (i.e., abnormally rapid growth of the sensation of sound intensity in the presence of hearing loss). When needed, contralateral masking was applied in an automated manner in line with audiometry guidelines for calculation of the efficacy and no-overmasking criteria [13]. In order to obtain the full psychometric function, the minimum and maximum intelligibility scores, as well as 3–5 additional PLs in between the minimum and maximum PLs were tested.

### 2.6. Speech Audiometry in Noise with Dodelé Logatoms

An initial Dodelé logatom list from the 5 Dodelé logatom lists (17 words of 3 phonemes each) was randomly chosen and played at 60 dB SPL in quiet for familiarization of the subject with the task. Next, in order to obtain the full psychometric function, different signal-to-noise-ratio (SNR) conditions were presented with the stimuli presentation level remaining constant at 60 dB SPL (considered as “normal” conversation level):

- 10 dB SNR = masking noise presented at 50 dB SPL;
- 5 dB SNR = masking noise presented at 55 dB SPL;
- 0 dB SNR = masking noise presented at 60 dB SPL;
−5 dB SNR = masking noise presented at 65 dB SPL;
−10 dB SNR = masking noise presented at 70 dB SPL;
−15 dB SNR = masking noise presented at 75 dB SPL;
−20 dB SNR = masking noise presented at 80 dB SPL.

For NH subjects, all SNR conditions were tested in the following order: 0 dB SNR, −5 dB SNR, −10 dB SNR, −15 dB SNR, −20 dB SNR, 5 dB SNR, and 10 dB SNR. For HI subjects, the SNR conditions tested were 0 dB SNR, −5 dB SNR, −10 dB SNR, 5 dB SNR, and 10 dB SNR. The masking noise was played around 1–3 s prior to the test word and the subject was instructed to repeat the word when they saw a green light on a monitor. This ensured that no sound was presented when the subject repeated the word (during a period of 3 s). If the score obtained after the first 7 words of a given list was less than 15%, the list was stopped, the subject’s score was saved and the next audiometry point (i.e., the 5 dB higher masking noise) was automatically set at 0% of correctly perceived phonemes, without being tested.

### 2.7. Manual Scoring of Subjects’ Verbal Responses in Quiet and in Noise by an Experimenter

For the entire test duration, the experimenter (i.e., one of the three expert hearing professionals) was seated in front of the subject in a sound-proof audiometric booth. The experimenter listened to and manually scored the subject’s verbal responses “live”—i.e., during the speech audiometry test. The scoring was performed at the phonemic level by the experimenter and manually entered into an Excel spreadsheet. Next, for each list, a global score was calculated from the phonemic scores (i.e., mean % score of correctly repeated phonemes over all phonemes tested). Experimenters were instructed not to speak, nor provide any feedback to the subject during the tests.

### 2.8. Automated Scoring of Subjects’ Verbal Responses in Quiet and in Noise by the ASR System

For the entire test duration, the audiometry software iAudiogram^®^ (v1; My Medical Assistant SAS, Reims, France) recorded the subject’s verbal responses through a USB microphone (LJU02, Shenzhen United Technology, Guangdong, China) and the ASR system developed (see Section 2.1) analyzed the subject’s verbal responses “live”—i.e., during the speech audiometry test. Scoring was performed in an automated manner by the ASR system at the phonemic level. After the test, all phonemic scores were transferred to the Excel spreadsheet. As for the manual scoring, for each list, a global score was calculated from the phonemic scores recorded.

### 2.9. Statistics

All group-level statistical tests and effect size calculations were performed using JMP Pro 14.0 on a Mac platform. To compare the manual scoring by human experts and the automated scoring by ASR, we computed both the raw and absolute differences. The raw difference corresponds to the “Automated score” minus the “Manual score“, and may contain both positive and negative values. In contrast, the absolute difference corresponds to the absolute value of the “Automated score” minus the “Manual score”. Hence, only positive values are present. Computing raw differences can be misleading as underestimations and overestimations of the ASR (i.e., positive and negative difference values) partially cancel each other out when computing a mean difference value. Absolute differences do not cancel each other and provide a meaningful value of the mean difference between the “Automated score” and the “Manual score”. Prior to performing statistical analyses, the Shapiro–Wilk test of normality was performed for all datasets. Non-normally distributed data were examined using non-parametric tests. Pairwise comparisons were carried out using the Steel–Dwass Method for non-parametric comparisons. To compare more than 2 groups, one-way ANOVA rank tests or Kruskal–Wallis H tests were used. Normally distributed data were examined using Student *t*-tests and ANOVAs, as described in Section 3. For post-hoc multiple comparisons analyses, alpha values were Holm–Bonferroni-corrected. For all analyses, data from the three expert hearing professionals were merged.

## 3. Results

### 3.1. Speech Audiometry in Quiet with Lafon’s Cochlear Lists

#### 3.1.1. Comparison of Manual and Automated Scoring for Each Lafon’s Cochlear Lists

For each of the 20 Lafon’s cochlear lists, we compared the raw and absolute differences in % intelligibility scores between the manual scoring by human experts and the automated scoring by ASR. Table 1 shows the difference values for each of the lists (i.e., errors made by the ASR as compared to the manual score by human experts). A mixed-model ANOVA revealed no significant main effect of the type of scoring (manual vs automated for all words of all lists; F(1, 19) = 0.82, *p* = 0.366). Post hoc comparisons confirmed no significant differences for all words of each of the 20 lists (*p* > 0.05).

#### 3.1.2. Comparison of Manual and Automated Scoring for Lafon’s Cochlear Lists as a Function of Hearing Status

**Figure 1:**
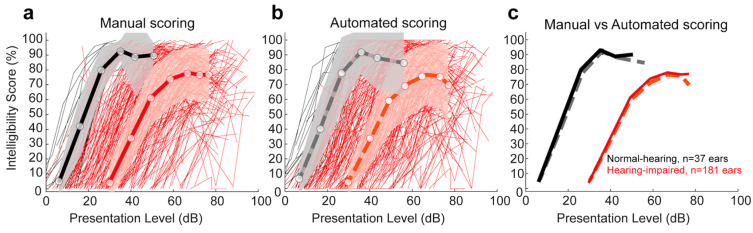
Comparison of manual and automated scoring of speech audiometry in quiet with Lafon’s cochlear lists: (**a**) Mean and std of intelligibility scores with manual scoring (black lines: NH; red lines: HI; thin lines represent individual ears tested). (**b**) Mean and std of intelligibility scores with automated scoring with ASR. (**c**) Comparison of the means with manual (full lines) and automated scoring (dashed lines).

For all subjects tested, we compared the raw and absolute differences in % intelligibility between the manual scoring by human experts and the automated scoring by ASR. Figure 1 shows the intelligibility scores of all normal-hearing subjects (NH, in black; thin lines show individual ears, thick line shows overall mean; shaded area represents ± 1 std) and all hearing-impaired subjects (HI, in red). Manual scoring data are shown with full lines in Figure 1a and ASR automated scoring data are shown with dashed lines in Figure 1b. For comparison purposes, the mean values of both manual and automated scoring are shown in Figure 1c. Supplementary Figure S1 (sections a and b) shows all individual raw and absolute differences. Table 2 provides the mean (± std) of the raw and absolute difference values for all NH and HI subjects. No significant effect of the type of scoring (manual vs automated) was found for NH subjects (mixed-model ANOVA; F(1, 72) = 0.17, *p* = 0.676), nor for the HI subjects (F(1, 362) = 0.44, *p* = 0.506).

#### 3.1.3. Comparison of Manual and Automated 50% and Maximum Intelligibility Scores for Lafon’s Cochlear Lists

Next, we examined two metrics extracted from the psychometric function of each subject: (1) the 50% intelligibility score, and (2) the maximum intelligibility score. The 50% intelligibility score obtained following manual scoring for all subjects differed from the one obtained following automated scoring by a value of −2.42 dB ± 11.92 (absolute difference= 4.11 ± 11.53). The maximum intelligibility obtained following manual scoring differed from the one obtained following automated scoring by a value of −1.92% ± 14.49 (absolute difference= 7.33 ± 12.65). No significant difference was found between 50% intelligibility scores obtained by manual and automated scoring (Wilcoxon two-sample test, *p* = 0.138). Similarly, no significant difference was found between maximum intelligibility scores obtained by manual and automated scoring (*p* = 0.098).

**Figure 2:**
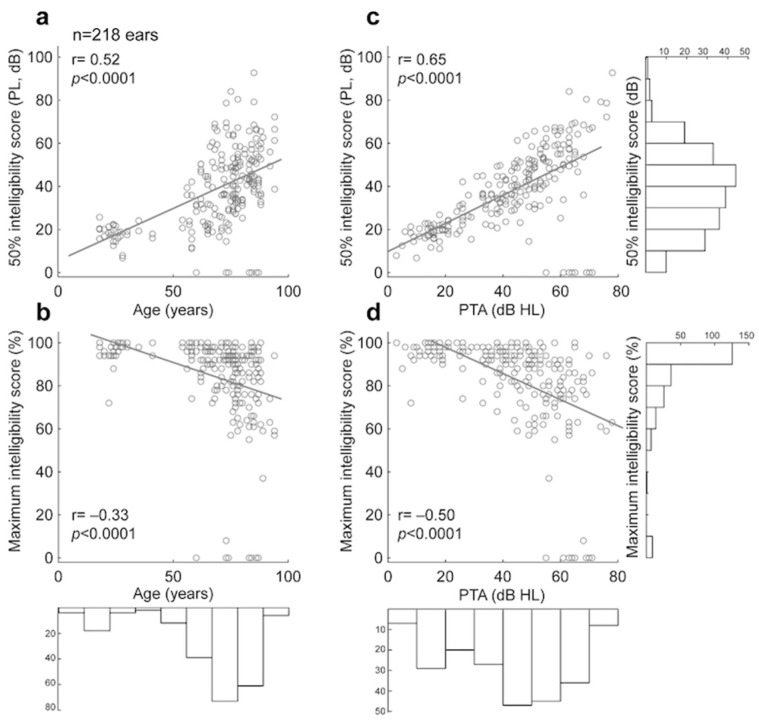
Correlations and histograms of age, PTA, 50% intelligibility score, and maximum intelligibility score for speech audiometry in quiet: (**a**) 50% intelligibility score obtained from manual scoring plotted against Age for all subjects. (**b**) 50% intelligibility score obtained from manual scoring plotted against PTA for all subjects. (**c**) Maximum intelligibility score obtained from manual scoring plotted against Age for all subjects. (**d**) Maximum intelligibility score obtained from manual scoring plotted against PTA for all subjects.

Significant correlations (Pearson’s correlation, *p* < 0.0001) were found when comparing both age and PTA against 50% intelligibility scores and maximum intelligibility scores (both manually scored; see Figure 2). When controlling for the effect of PTA, age was no longer significantly correlated with 50% intelligibility scores and maximum intelligibility scores (partial correlations: *p* = 0.14; *p* = 0.44, respectively). In contrast, when controlling for the effect of age, PTA remained significantly correlated with 50% intelligibility scores and maximum intelligibility scores (partial correlations: *p* < 0.0001 for both).

#### 3.1.4. Convergence of Scores for Each of Lafon’s Cochlear Lists

Measuring the full psychometric function can be time-consuming, especially if all 17 words present in a given speech list are tested for each stimulus presentation level (PL). Nevertheless, in contrast to only estimating the 50% intelligibility score, the full psychometric function offers several advantages: richer data, information about performance plateaus (maximum intelligibility score) and slope of the function, and informs hearing professionals about hearing-aid or implant fittings (gain adjustments can be optimized). The main aim of this section is to analyze whether all 17 words of a given list need to be tested at each stimulus presentation level (PL) or whether fewer words (per PL) can be used to save time.

Here, we only examined data from the manual scoring method for all subjects and all lists. For each ear of each subject, we looked at the difference in score between the final word of the given list (i.e., word 17) and the other 16 words of the given list. Table 3 shows the difference in scores averaged across all tested subjects’ ears (all ears tested; n = 218, middle column), and across all tested lists (all ears tested with each given list; n = 20, last column). These results show that conservatively (i.e., when adding mean to the std), a <10% difference in scores (compared to the final score) is obtained at word 7 (per subject’s ear average) and at word 5 (per list average). A <5% difference in scores is obtained at word 12 (per subject’s ear average) and at word 11 (per list average).

#### 3.1.5. Test–Retest Reliability of Manual and Automated Scoring of Lafon’s Cochlear Lists

A subset of 20 subjects (6 men, 14 women; 49.3 ± 21.47 years old) was tested twice, and the test–retest difference values are shown in Table 4. The two manual scorings were performed by the same experimenter in exactly the same testing conditions and were spaced by a maximum of 1 month. No notable otological history was identified during this period. No significant difference was found between the test–retest manual scoring values (Kruskal–Wallis H test, X^2^(1) = 2.59, *p* = 0.107). Similarly, no significant difference was found between the test–retest automated scoring values (X^2^ (1) = 3.33, *p* = 0.068).

In line with the above results, the test–retest 50% intelligibility scores did not differ significantly for the two scoring methods (manual scoring: *p* = 0.136; automated scoring: *p* = 0.093). Similarly, the test–retest maximum intelligibility scores did not differ significantly for the two scoring methods (manual scoring: *p* = 0.722; automated scoring: *p* = 0.345).

### 3.2. Speech Audiometry in Noise with Dodelé Logatoms

#### 3.2.1. Comparison of Manual and Automated Scoring for Each Dodelé Logatom List

For all five Dodelé Logatom lists and the different signal-to-noise (SNR) conditions tested, ranging from −20 to +10 SNR (see Section 2.6), we compared the raw and absolute differences in % intelligibility between the manual scoring by human experts and the automated scoring by ASR. Table 5 shows the difference values for each of the lists. A mixed-model ANOVA revealed no significant main effect of the type of scoring (manual vs automated for all words of all lists; F(1, 496) = 0.27, *p* = 0.606). Post hoc comparisons confirmed no significant differences for all words of each of the 5 lists (*p* > 0.05).

#### 3.2.2. Comparison of Manual and Automated Scoring for Dodelé Logatom Lists as a Function of Hearing Status

**Figure 3:**
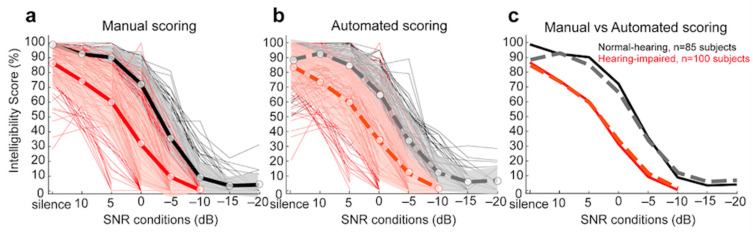
Comparison of manual and automated scoring of speech audiometry in noise with Dodelé logatom lists: (**a**) Mean and std of intelligibility scores with manual scoring (black lines: NH; red lines: HI; thin lines represent individual ears). (**b**) Mean and std of intelligibility scores with automated scoring with ASR. (**c**) Comparison of the means with manual (full lines) and automated scoring (dashed lines).

For all subjects tested, we compared the raw and absolute differences in % intelligibility between the manual scoring by human experts and the automated scoring by ASR. Figure 3 shows the intelligibility scores of all NH subjects (in black; thin lines show individual ears, thick line shows overall mean; shaded area represents ± 1 std) and all HI subjects (in red). Manual scoring data are shown with full lines in Figure 3a and ASR automated scoring data are shown with dashed lines in Figure 3b. For comparison purposes, the mean values of both manual and automated scoring are shown in Figure 3c. Supplementary Figure S1 (sections c and d) shows all individual raw and absolute differences. Table 6 provides the mean (± std) of the raw and absolute difference values for all NH and HI subjects for all SNR conditions tested. No significant effect of the type of scoring (manual vs automated) was found for NH subjects (mixed-model ANOVA; F(1, 165) = 3.49, *p* = 0.063), nor for the HI subjects (F(1, 198) = 0.01, *p* = 0.911) for all SNR conditions tested.

#### 3.2.3. Comparison of Manual and Automated 50% and Maximum Intelligibility Scores for Dodelé Logatom Lists

As for speech audiometry in quiet, we examined two metrics extracted from the psychometric function of each subject: (1) the 50% intelligibility score, and (2) the maximum intelligibility score. The 50% intelligibility score obtained following manual scoring for all subjects differed from the one obtained following automated scoring by a value of 0.29 dB SNR ± 1.28 (absolute difference= 0.81 ± 1.03). The maximum intelligibility obtained following manual scoring differed from the one obtained following automated scoring by a value of −4.90% ± 15.00 (absolute difference= 7.18 ± 14.05). No significant difference was found between 50% intelligibility scores obtained by manual and automated scoring (Wilcoxon two-sample test, *p* = 0.458). Similarly, no significant difference was found between maximum intelligibility scores obtained by manual and automated scoring for all SNR conditions (*p* = 0.075).

**Figure 4:**
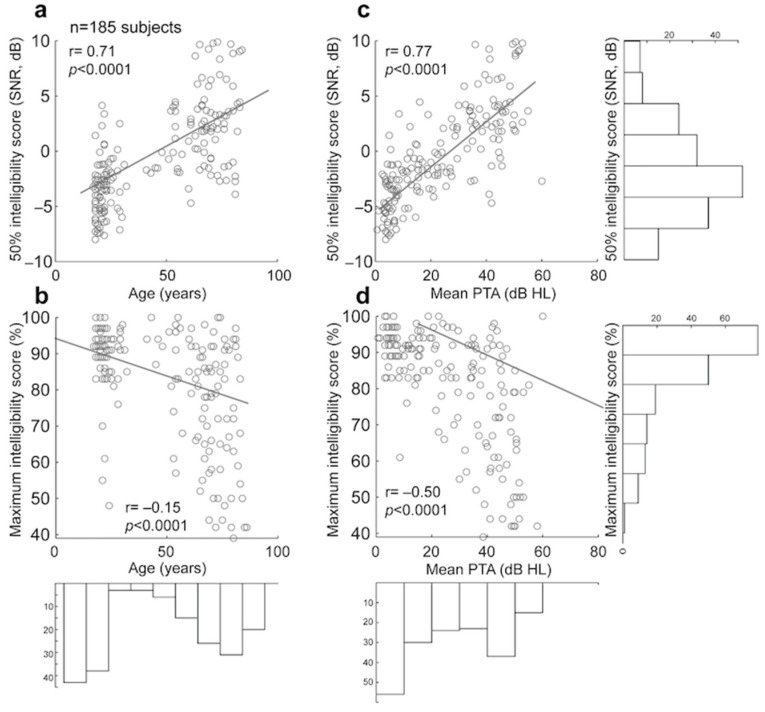
Correlations and histograms of age, PTA, 50% intelligibility score, and maximum intelligibility score for speech audiometry in noise: (**a**) 50% intelligibility score obtained from manual scoring plotted against Age for all subjects. (**b**) 50% intelligibility score obtained from manual scoring plotted against PTA for all subjects. (**c**) Maximum intelligibility score obtained from manual scoring plotted against Age for all subjects. (**d**) Maximum intelligibility score obtained from manual scoring plotted against PTA for all subjects.

Significant correlations (Pearson’s correlation, *p* < 0.0001) were found when comparing both age and PTA against 50% intelligibility scores and maximum intelligibility scores (both manually scored; see Figure 4). When controlling for the effect of PTA, age was still significantly correlated with 50% intelligibility scores, but not with maximum intelligibility scores (partial correlations: *p* = 0.01; *p* = 0.69, respectively). When controlling for the effect of age, PTA remained significantly correlated with 50% intelligibility scores and maximum intelligibility scores (partial correlations: *p* < 0.0001 for both). These results suggest that both PTA and age can accurately predict the 50% intelligibility scores in noise and only PTA can accurately predict the maximum intelligibility scores in noise.

#### 3.2.4. Convergence of Scores for Each of Dodelé Logatom Lists

The main aim of this section is to analyze whether all 17 words of a given list need to be tested at each SNR or whether fewer words per SNR can be tested to save time. Hence, we only examined data from the manual scoring method for all subjects and all lists. For each individual subject tested, we looked at the difference in score between the final word of the given list (i.e., word 17) and the other 16 words of the given list. Table 7 below shows the difference in scores averaged across all tested subjects (n = 185, middle column), and across all tested lists (n = 5, last column). These results show that conservatively (i.e., when adding mean to the std), a <10% difference in scores (compared to the final score) is obtained at word 7 (per subject average and per list average). A <5% difference in scores is obtained at word 12 (per subject average and per list average).

#### 3.2.5. Test–Retest Reliability of Manual and Automated Scoring of Dodelé Logatom Lists

A subset of 111 subjects (57 NH, 54 HI; 54.3 ± 20.14 years old) was tested twice, and the test–retest difference values are shown in Table 8. The two manual scoring tests were performed by the same experimenter in exactly the same testing conditions and were spaced by a maximum of 1 month. No notable otological history was identified during this period. No significant difference was found between the test–retest manual scoring values (mixed-model ANOVA, F(1, 50) = 0.76, *p* = 0.386). Similarly, no significant difference was found between the test–retest automated scoring values (F(1, 50) = 0.04, *p* = 0.851). 

In line with the above results, the test–retest 50% intelligibility scores did not differ significantly for the two scoring methods (manual scoring: *p* = 0.060; automated scoring: *p* = 0.064). Similarly, the test–retest maximum intelligibility scores did not differ significantly for the two scoring methods (manual scoring: *p* = 0.359; automated scoring: *p* = 0.488).

## 4. Discussion

ASR systems have rapidly become integral to our daily lives (e.g., Apple Siri or Amazon Alexa), and are increasingly supporting healthcare providers and patients. Examples include automated transcription of medical reports (e.g., [58]), automated speech training (e.g., [59]), estimation of speech test performance (e.g., [60]), and automated speech audiometry scoring ([30,33,39,41,42], review: [44]). In this study, we developed an ASR system (see Methods, Section 2.1) to automate speech audiometry with Lafon’s Cochlear lists for speech-in-quiet tests and Dodelé logatoms for speech-in-noise tests. The ASR system was trained and tested on anonymized speech audiometry responses recorded from a large patient cohort. These audio recordings were manually labeled by hearing professionals and served as training and testing datasets for the ASR system. The initial pre-validation of the ASR system demonstrated strong performance, with 90.8% correct phonemic identification and 87.75% correct word identification. We further evaluated the ASR performance (i.e., accuracy: comparison with manual scoring) and reliability (i.e., test–retest) in real-world clinical conditions. To do so, we tested a large sample of subjects with a wide age range and different types of hearing status, including varying degrees of hearing loss (see Methods, Section 2.2).

For speech-in-quiet audiometry, our results confirmed the ASR performance across each of the 20 Lafon’s cochlear lists (see Table 1), and for both NH and HI subjects (see Figure 1). No significant difference was found between the two types of scoring methods (manual by human experts vs automated by ASR; NH: *p* = 0.676; HI: *p* = 0.506). Similarly, no significant difference was found between the two scoring methods for the 50% intelligibility score (mean absolute difference = 4.11 dB; *p* = 0.138) and the maximum intelligibility score (mean absolute difference = 7.33%; *p* = 0.098). The test–retest reliability showed similar differences for the two scoring methods (Table 4). Importantly, the 50% intelligibility score differed by less than 5 dB for the test–retest automated scoring method (mean absolute difference = 3.44 dB). It is important to note that the manual test–retest reliability measured here (mean absolute difference = 5.95 dB) may be slightly underestimated as the experimenters did not have to manually determine the stimulus presentation levels but only scored patient responses. All stimulus presentation levels were automatically chosen (see Methods, Section 2.5 and Section 2.6) by the audiometry software iAudiogram^®^ (v1, My Medical Assistant SAS, Reims, France).

For speech audiometry in noise, our results confirmed the ASR performance across each of the five Dodelé Logatom lists (see Table 5), and for both NH and HI subjects (see Figure 3). No significant difference was found between the two types of scoring methods (manual by human experts vs automated by ASR; NH: *p* = 0.063; HI: *p* = 0.911). In line with the above results, no significant difference was found between the two types of scoring methods for the 50% intelligibility score (mean absolute difference = 0.81 dB SNR; *p* = 0.458) and the maximum intelligibility score (mean absolute difference = 7.18%; *p* = 0.075). Overall (for all lists and all subjects), the phonemic error rate was estimated at 7.26% ± 8.76 for NH subjects and at 6.20% ± 7.09 for HI subjects. These results are in line with a previous study [42], in which the authors estimated a word error rate for NH subjects at 5.0% ± 8.8, and with [30,61,62] in which a “sentence” accuracy of 90.7% was measured in NH subjects. In addition, for all subjects, the manual and automated 50% intelligibility score differed by less than 1 dB SNR (mean absolute difference = 0.81 dB SNR ± 1.03). This is comparable to data analyzed in [41] in which a mean difference of 1.40 dB SNR ± 2.63 was found. Finally, the test–retest reliability showed similar differences for the two scoring methods (Table 8). Importantly, the 50% intelligibility score differed by less than 2 dB SNR for the test–retest automated scoring method (mean absolute difference = 1.51 dB SNR ± 1.25). 

Next, for all speech lists tested, we examined how quickly a subject’s responses converged to the final score (see Table 3 and Table 7). With phonetic-level scoring, a less than 10% difference was found between the score computed at word 7 compared to the final score—computed at word 17. These results suggest that depending on the desired accuracy, fewer words (e.g., 5 or 7 words instead of 17) may be used in each list and for each presentation condition (dB or dB SNR) in order to save time.

ASR systems for automating speech audiometry offer several key advantages. First, by eliminating the need for human supervision and manual scoring of patient responses, ASR systems free up medical professional’s time. This enables speech tests to be conducted more frequently and systematically as part of comprehensive hearing assessments [19,26,63]. Second, automated scoring can facilitate the assessment of baseline curves for NH listeners across various speech materials available and testing conditions (e.g., headphones vs free-field, different number of loudspeakers, positioning of loudspeakers). Third, ASR systems can be adapted to accommodate a broader and more diverse patient population, including individuals with strong accents, or those who are non-native speakers, e.g., [64]. While manual scoring is generally robust, well-trained ASR systems—using sufficiently diverse and high-quality training datasets—may achieve even greater accuracy in identifying responses from non-native speakers or those with strong accents. Future studies should focus on evaluating the suitability and performance of current ASR systems for these ‘special’ populations to ensure inclusivity and effectiveness.

Deep neural networks aim to learn in ways analogous to how humans and animals learn (e.g., [65]; but see [66]). Artificial neural networks were originally designed by mimicking the computational principles of the nervous system [67]. In this study, we used the wav2vec 2.0 model [50], a recently proposed and pre-trained, self-supervised network for speech representation learning [68] that we fine-tuned using a large dataset of speech audiometry data to perform phonetic-level recognition of words. This is akin to how a human child learns to speak (and recognize words) after listening to, and “being trained” on a vast amount of speech sounds during development (e.g., [69]). More precisely, phoneme awareness in children seems to be a good predictor of reading skills [70].

The key difference between the wav2vec 2.0 model used here [50] and traditional methods like deep neural network–hidden Markov model (DNN-HMM) [30,33,39,41] and Kaldi [42] lies in their approach to speech recognition. DNN-HMM systems and Kaldi use a hybrid architecture requiring separate acoustic, pronunciation, and language models, along with carefully prepared transcribed training datasets. In contrast, the wav2vec 2.0 model represents an end-to-end approach that learns directly from raw audio waveforms using self-supervised learning. More precisely, the wav2vec 2.0 model is pre-trained on unlabeled speech data to learn acoustic representations, then it is fine-tuned on a relatively small amount of labeled data. Additionally, the wav2vec 2.0 model eliminates the need for hand-crafted features like spectrograms or mel-frequency cepstral coefficients that are typically required in traditional approaches, as it learns useful representations directly from raw audio waveforms.

Moreover, artificial neural networks are designed to identify optimal solutions for specific problems they are tasked to solve. Here, the deep neural network evaluates its outputs against the manually label inputs to ensure that the outputs maximally represent the inputs. Conceptually, this approach is—in part—similar to the operations performed by neuronal ensemble dynamics during associative learning in the central nervous system, where repeated and timely pairings of sensory inputs with motor outputs form strong input-output associations using mechanisms of Hebbian plasticity (e.g., [71,72,73,74]). In the recent years, the application of deep neural networks in the auditory and audiology fields has become increasingly successful and popular—particularly, in the hearing aids field (e.g., [75]). More precisely, advanced neural networks have been created to process sound and provide exceptional speech clarity in a range of listening environments for individuals with hearing impairments (e.g., Oticon MoreTM; Phonak Infinio Sphere [76,77,78]). These hearing-aid technologies illustrate the versatility and effectiveness of artificial neural networks in addressing challenges within the auditory and audiology fields (see review [79]).

## 5. Conclusions

In summary, the ASR system developed here is accurate and reliable for both research and clinical use on native French speakers without strong regional accents. No statistical difference was found when evaluating the accuracy of the ASR system as compared to manual scoring by human experts for both speech-in-quiet and speech-in-noise tests. Furthermore, the test–retest reliability results were consistent (the 50% intelligibility score differed by less than 2 dB SNR for speech-in-noise), showing the ASR system’s reliability.

Future studies should investigate whether the current deep neural network can generalize to non-native French speakers and other French-language speech materials and assess its performance with a larger group of HI subjects and cochlear implant users. Here, relatively few HI listeners with severe hearing loss were tested in the speech-in-silent condition and no HI listener with severe hearing loss was tested in the speech-in-noise condition. Moreover, no cochlear implant user was tested in either condition. It is important to note that severe hearing loss and profound deafness can result in disordered speech [80], which may lead to poor ASR performance [81,82]. Hence, the ASR system’s accuracy and reliability should be evaluated in future studies involving patients with severe hearing loss, cochlear implant users, and those with hearing-aid fittings (e.g., [83]).

## 6. Patents

N.W. has a patent pending on the technology described in the manuscript.

## Figures and Tables

**Table 1:** Comparison of manual and automated scoring for each Lafon’s cochlear lists (mean ± 1 std).

Automated vs. Manual Scoring (Number of Ears Tested for Each Comparison)	Raw Differences (%)	Absolute Differences (%)	Post Hoc Comparisons (*p*-Value)
Lafon’s Cochlear List 1 (n = 58 ears)	3.09 ± 15.52	8.40 ± 13.37	0.601
List 2 (n = 59 ears)	1.98 ± 16.01	9.24 ± 13.17	0.728
List 3 (n = 61 ears)	0.05 ± 8.90	4.34 ± 7.75	0.993
List 4 (n = 63 ears)	2.78 ± 6.92	4.30 ± 6.07	0.676
List 5 (n = 66 ears)	0.74 ± 7.27	4.35 ± 5.85	0.908
List 6 (n = 67 ears)	−1.03 ± 7.43	4.31 ± 6.12	0.869
List 7 (n = 61 ears)	1.21 ± 6.70	5.05 ± 4.53	0.812
List 8 (n = 67 ears)	−1.65 ± 12.65	6.60 ± 10.89	0.766
List 9 (n = 27 ears)	0.48 ± 8.37	5.00 ± 6.66	0.891
List 10 (n = 70 ears)	2.16 ± 16.82	6.49 ± 15.64	0.448
List 11 (n = 69 ears)	−1.21 ± 11.75	6.31 ± 9.95	0.985
List 12 (n = 65 ears)	−1.94 ± 9.77	5.66 ± 8.17	0.755
List 13 (n = 66 ears)	−2.38 ± 12.34	6.48 ± 10.74	0.645
List 14 (n = 62 ears)	1.06 ± 15.75	7.45 ± 13.88	0.853
List 15 (n = 64 ears)	0.06 ± 7.25	4.59 ± 5.58	0.992
List 16 (n = 61 ears)	−4.23 ± 13.97	7.54 ± 12.47	0.474
List 17 (n = 62 ears)	−2.47 ± 11.79	6.47 ± 10.13	0.669
List 18 (n = 62 ears)	0.56 ± 16.18	7.64 ± 14.24	0.917
List 19 (n = 61 ears)	−0.75 ± 9.27	6.44 ± 6.65	0.910
List 20 (n = 58 ears)	−0.14 ± 13.97	7.79 ± 11.55	0.557
All lists	**−0.12 ± 12.03**	**6.22 ± 10.30**	**0.366**

**Table 2:** Mean difference (±1 std) between manual and automated scoring for Lafon’s cochlear list (see also Supplementary Figure S1).

Automated vs. Manual Scoring	Raw Difference (%)	Absolute Difference (%)
Normal hearing (NH) n = 37 ears	0.93 ± 6.44	4.13 ± 5.02
Hearing impaired (HI) n = 181 ears	−0.91 ± 9.88	5.57 ± 8.01

**Table 3:** Comparison of scores for each word (e.g., word #1) to final word (word #17) for all subjects and all Lafon’s cochlear lists.

Difference in Score of Given Word# Compared to Score for Word #17 (mean ± 1 std)	Absolute Differences (%) (per Ear Average)	Absolute Differences (%) (per List Average)
First word of given list: word #1	23.88 ± 7.02	24.19 ± 4.84
word #2	15.24 ± 4.75	15.40 ± 2.18
word #3	11.58 ± 3.81	11.63 ± 1.62
word #4	9.66 ± 3.47	9.68 ± 1.82
word #5	7.96 ± 2.83	8.02 ± 1.32
word #6	6.99 ± 2.51	7.09 ± 1.03
word #7	6.29 ± 2.56	6.38 ± 1.33
word #8	5.75 ± 2.30	5.87 ± 0.92
word #9	5.06 ± 1.91	5.17 ± 0.89
word #10	4.50 ± 1.67	4.59 ± 0.60
word #11	3.96 ± 1.48	4.05 ± 0.46
word #12	3.42 ± 1.25	3.47 ± 0.36
word #13	2.80 ± 1.00	2.86 ± 0.42
word #14	2.21 ± 0.86	2.29 ± 0.58
word #15	1.67 ± 0.68	1.67 ± 0.36
word #16	1.13 ± 0.59	1.11 ± 0.18
Last word of given list: word #17	0	0

**Table 4:** Mean test–retest differences for manual and automated scoring for speech audiometry in quiet.

Test vs. Retest (n = 40 Ears)		Raw Differences	Absolute Differences
Manual Scoring	All words tested (%)	−2.20 ± 13.03	8.31 ± 10.23
	50% intelligibility score (dB)	−3.72 ± 15.81	5.95 ± 15.08
	Maximum intelligibility score (%)	−2.00 ± 7.68	4.00 ± 6.81
Automated Scoring	All words tested (%)	−4.08 ± 17.22	10.05 ± 14.53
	50% intelligibility score (dB)	0.29 ± 6.06	3.44 ± 4.29
	Maximum intelligibility score (%)	−1.94 ± 6.51	4.05 ± 5.40

**Table 5:** Comparison of manual and automated scoring for each Dodelé logatom list (mean ± 1 std).

Automated vs. Manual Scoring (All Subjects Tested for Each Comparison, n = 185)	Raw Differences (%)	Absolute Differences (%)	Post Hoc Comparisons (*p*-Value)
Dodelé Logatoms, List 1 (n = 185 subjects)	−1.09 ± 9.30	5.04 ± 7.87	0.225
List 2	−4.34 ± 11.14	9.18 ± 9.89	0.505
List 3	−6.51 ± 9.75	8.90 ± 7.57	0.055
List 4	−3.98 ± 10.27	7.62 ± 7.91	0.222
List 5	2.98 ± 6.51	5.20 ± 4.87	0.297

**Table 6:** Mean difference (± 1 std) between manual and automated scoring for Dodelé logatom lists (see also Supplementary Figure S1).

Automated vs. Manual Scoring	Raw Difference (%)	Absolute Difference (%)
Normal hearing (NH) n = 85 subjects	−3.50 ± 10.83	7.26 ± 8.76
Hearing impaired (HI) n = 100 subjects	−1.56 ± 9.30	6.20 ± 7.09

**Table 7:** Comparison of scores for each word (e.g., word #1) to final word (word #17) for all subjecs and all Dodelé logatom list.

Difference in Score of Given Word# Compared to Score for Word #17 (mean ± std)	Absolute Differences (%) (per Ear Average)	Absolute Differences (%) (per List Average)
First word of given list: word #1	29.45 ± 11.40	21.28 ± 0.74
word #2	16.15 ± 6.78	15.33 ± 1.18
word #3	11.49 ± 4.72	13.07 ± 1.09
word #4	9.69 ± 4.20	11.27 ± 1.12
word #5	8.18 ± 3.72	9.76 ± 1.29
word #6	7.11 ± 3.18	8.74 ± 0.81
word #7	6.37 ± 2.79	7.28 ± 0.64
word #8	5.32 ± 2.50	6.37 ± 0.50
word #9	4.60 ± 2.16	5.59 ± 0.28
word #10	4.08 ± 2.04	4.91 ± 0.62
word #11	3.62 ± 1.80	4.02 ± 0.52
word #12	2.83 ± 1.46	3.54 ± 0.42
word #13	2.52 ± 1.11	2.91 ± 0.36
word #14	1.95 ± 0.92	2.21 ± 0.32
word #15	1.59 ± 0.71	1.59 ± 0.26
word #16	0.94 ± 0.38	1.35 ± 0.44
Last word of given list: word #17	0	0

**Table 8:** Mean test–retest differences for manual and automated scoring for speech audiometry in noise.

Test vs. Retest (n = 111 Subjects)		Raw Differences	Absolute Differences
Manual Scoring	All words tested (%)	2.61 ± 9.03	5.94 ± 7.28
	50% intelligibility score (dB SNR)	−0.71 ± 1.37	1.18 ± 0.99
	Maximum intelligibility score (%)	1.33 ± 2.23	1.89 ± 1.75
Automated Scoring	All words tested (%)	1.81 ± 11.53	8.06 ± 8.45
	50% intelligibility score (dB SNR)	−0.64 ± 1.85	1.51 ± 1.25
	Maximum intelligibility score (%)	−2.06 ± 5.69	2.94 ± 4.51

## Data Availability

The original data presented in the study can be shared upon request to the last author: Nihaad Paraouty (paraouty@iaudiogram.com).

## Supplementary Materials

The following supporting information can be downloaded at: www.mdpi.com/article/10.3390/biology14020191/s1.

## Abbreviations

The following abbreviations are used in this manuscript:

ASR	Automatic Speech Recognition
AC	Air conduction
PL	Presentation Level
PTA	Pure-tone average
ML	Machine learning
SRT	Speech reception threshold
NH	Normal hearing
HI	Hearing impaired
DNN	Deep neural network
SNR	Signal-to-noise ratio
std	Standard deviation
HMM	Hidden Markov model
ENT	Ear–Nose–Throat specialist
