# Supplementary material for: Automating Speech Audiometry in Quiet and in Noise Using a Deep Neural Network
Source: Biology (Basel). 2025 Feb 12;14(2):191. doi: 10.3390/biology14020191 (PMC11851792; doi:10.3390/biology14020191)
Supplement: Supplementary file 1 [file biology-14-00191-s001.zip › biology-3433727-supplementary.pdf]

Supplementary Figure S1

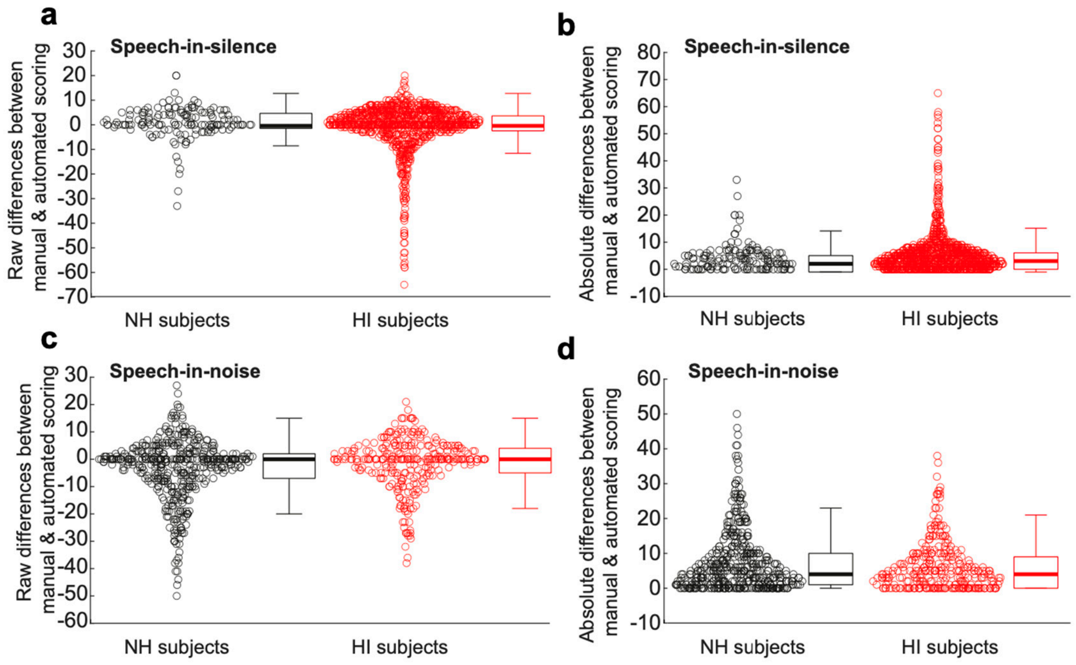

Supplementary Figure S1. Raw and absolute differences between manual and automated scoring: (**a**) (Left) Raw differences for all individual NH subjects' ears (black circles, n=37 ears) and all individual HI subjects' ears (red circles, n=181 ears) when comparing manual and automated scoring for speech audiometry in quiet with Lafon's Cochlear lists for all presentation levels tested. Mean (and std) difference values are provided in Table 2. (Right) Boxplots representing median (thick line), 25th and 75th percentiles (box) and nonoutlier minimum and maximum points; (**b**) Absolute differences for speech audiometry in quiet; (**c**) (Left) Raw differences for all individual NH subjects (black circles, n=85 subjects) and all individual HI subjects (red circles, n= 100 subjects) when comparing manual and automated scoring for speech audiometry in noise with Dodelé Logatoms lists for all SNR conditions tested. Mean (and std) difference values are provided in Table 6. (Right) Boxplots representing median (thick line), 25th and 75th percentiles (box) and nonoutlier minimum and maximum points. (**d**) Absolute differences for speech audiometry in noise.
